# Heat-Shock-Induced Removal of Transgenes Using the Gene-Deletor System in Hybrid Aspen (*Populus tremula* × *P. tremuloides*)

**DOI:** 10.3390/genes9100484

**Published:** 2018-10-08

**Authors:** Beibei Wang, Yan Zhang, Jian Zhao, Mingliang Dong, Jinfeng Zhang

**Affiliations:** 1 Beijing Advanced Innovation Center for Tree Breeding by Molecular Design, National Engineering Laboratory for Tree Breeding, Key Laboratory of Genetics and Breeding in Forest Tree and Ornamental Plants of Ministry of Education, Key Laboratory of Forest Trees and Ornamental Plants Biological Engineering of State Forestry Administration, College of Biological Sciences and Technology, Beijing Forestry University, Beijing 100083, China; wbb848172562@163.com (B.W.); zhangyan19890802@126.com (Y.Z.); zhaojian0703@bjfu.ed u.cn (J.Z.); dmllinxue@163.com (M.D.); 2 Beijing Academy of Forestry and Pomology Sciences, Beijing 100093, China

**Keywords:** gene-deletor system, *Populus*, heat shock, methylation, expression

## Abstract

To evaluate the efficacy of the gene-deletor system in aspen, we evaluated the system for foreign gene removal in a hybrid aspen clone, INRA 353-53 (*Populus tremula* × *P. tremuloides*). The recombinase flipping DNA (*FLP*) gene was under the control of the heat-inducible promoter of *Gmhsp17.6-L*, and the β-glucuronidase (*gusA*) gene which was under the control of the 35S promoter and were constructed using the gene-deletor system in the pCaLFGmFNLFG vector. Six transgenic plants and their sublines were heated at 42 °C for 8 h and gene deletion was verified by polymerase chain reaction (PCR). Three lines exhibited partial transgene deletion while the remaining three lines did not delete. Transgenic lines were evaluated by Southern-blot analyses, verifying that the six transgenic plant lines all had a single copy of transfer DNA (t-DNA). Two partial-deletion lines and two non-deletion lines were analysed for methylation and expression of promoter and recombinase. Hardly any methylation was detected in the *Gmhsp17.6-L* promoter or recombinase *FLP* gene sequences, however, the expression of the promoter and recombinase was increased significantly in the partial-deletion compared with the non-deletion line after heat-shock treatment. These results suggest that the excision efficiency had no direct relationship with methylation status of the *Gmhsp17.6-L* promoter and *FLP* recombinase, yet was affected by the expression of the *Gmhsp17.6-L* and *FLP* after heat-shock treatment.

## 1. Introduction

Since the first report of transgenic aspen [1], researchers have been using transgenic technology to improve plant biomass and increase resistance to insects, herbicides, and disease in aspen [2,3,4]. Transgenic plants can be used to address scientific questions by modification of flowering, hormone levels, and phytoremediation. However, transgenic plants may affect gene flow, increasing public concerns about environmental safety and thereby limiting the use of transgenic technology in trees.

Various strategies have been developed to remove marker or foreign genes in genetically modified plants. These methods include co-transformation of two transfer DNA (t-DNA) regions [5], homologous recombination [6], transposon-mediated reposition [7], site-specific recombination [8,9,10], and the gene-deletor system [11,12,13]. The gene-deletor system differs from other heat-inducible recombination systems [8,9,10] found in natural and has been constructed by a group [11,12]. It interacts with the recognition sit of Cre/*loxP* and Flp/*FRT* systems which were found in phage P1 and *Saccharmyces cerevisiae* respectively as the recognition sequence namely *loxP-FRT* for the recombinase. The system is more efficient in deleting foreign genes with flipping DNA (FLP) than with the combination of FLP and Cre, while the recognition site of FLP is just only *FRT* and of Cre is *loxP*. It has been used to delete all foreign genes in transgenic tobacco seeds and pollen using corresponding tissue-specific promoters [11] and to obtain marker-free transgenic tobacco under a cold-induced promoter [14]. Thus, the gene-deletor system provides the most effective method of addressing gene flow and food safety issues [13,15]. However, until now, the gene-deletor system has not been used successfully in aspen. 

Recombinase expression can be induced by tissue-specific promoters [12,16,17], chemical promoters [18,19,20] or heat-shock promoters [21,22,23,24], which may be the simplest and most frequently used promoters. The soybean *Gmhsp17.6-L* promoter is a heat-shock promoter that has been successfully used to induce flowering in aspens [25]; however, there is no study on the use of the gene-deletor system controlled by the *Gmhsp17.6-L* promoter in aspen.

In this study, we evaluated the feasibility of using the gene-deletor system with the *Gmhsp17.6-L* promoter in transgenic aspen. Further, we analysed the efficiency of foreign gene elimination and the factors influencing the efficiency thereof in transgenic aspen.

## 2. Materials and Methods

### 2.1. Vector Construction

**Figure 1:**
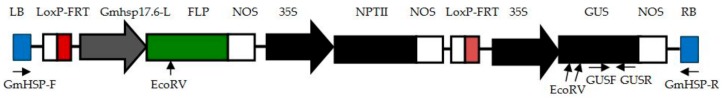
Schematic representation of the pCaLFGmFNLFG vector used for plant transformation. *Flipping DNA* (*FLP*) transcription is induced by the heat-shock promoter *Gmhsp17.6-L* from soybean. The *GUS* and *NPTII* genes are under the control of the CaMV 35S promoter. LB: Left border, *LoxP-FRT*: Fusion recognition sites, *Gmhsp17.6-L:* A heat-shock-inducible promoter from soybean, NOS: Polyadenylation sequence of the nopaline synthase gene, 35S: CaMV 35S promoter, GUS: Glucuronidase gene, NPTII: Neomycin phosphotransferase gene, RB: Right border, GmHSP-F, GUSF, GUSR, GmHSP-R: Primers binding sites, *Eco*RV: Restriction enzyme sites.

The pGM626-ZMF-ABt vector was provided by the Institute of Agro-Biotechnology (Guizhou University, China) and the pCaLFGmFGNLF vector is stored in our laboratory. The pCaLFGmFNLFG vector was constructed as follows: the NOS and RB sequences were amplified from the pGM626-ZMF-ABt vector using the III-NOS-FF/III-NOS-RR and III-RB-FF/III-RB-R primers (Table S1) and then ligated to the NOS-RB sequence generated by overlap polymerase chain reaction (PCR) via the KpnI and SphI restriction sites. The nopaline synthase and right border (NOS-RB) sequence with the KpnI and SphI sites was ligated into the pCaLFGmFGNLF vector to obtain pLF-Gmhsp-FLP-35S-GUS-NOS-RB-LF. XhoI-FLP–NOS and NPTII-NOS-LoxP/*FRT* were amplified from the pCaLFGmFGNLF vector using the Gmhsp-9/III-XhoI-R and III-NPT-FF/III-LOXP-RR primers (Table S1), and the 35S sequence was amplified from the pGM626-ZMF-ABt vector using the III-35S-FFF/III-35S-RR primers (Table S1). Xho I-35s-NPTII-NOS-loxP/*FRT* was obtained by ligating the pGmhsp-gus-XhoI, 35S and NPTII-NOS -loxP sequences. The XhoI-35s-NPTII–NOS-loxP sequence was ligated to pLF-Gmhsp-FLP-35S-GUS-NOS-RB-LF to obtain the pCaLFGmFNLFG vector (Figure 1).

### 2.2. Verification of the Transformation Vector

**Figure 2:**
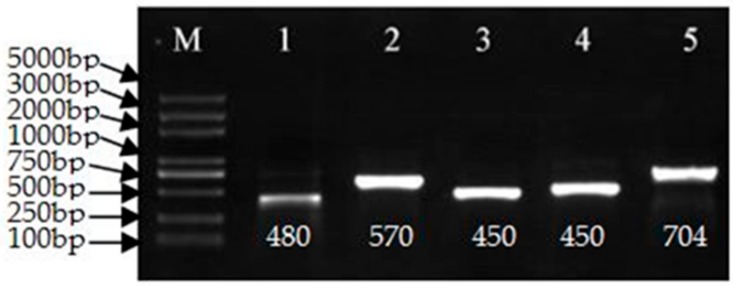
Polymerase chain reaction (PCR) confirmation of the pCaLFGmFNLFG vector. M: Marker 5000. PCR amplicons obtained using the following primer pairs: 1, GMHSP-F/GMHSP-R; 2, F3t/R3t; 3, Fmid/Rmid; 4, GUS-nptII-F/ GUS-nptII-R; and 5, FLP-35SF/ FLP-35SR. Bp: base pair.

The pCaLFGmFNLFG vector was evaluated by PCR using the primers listed in Table S2 and sequenced. Electrophoresis of the PCR products confirmed the expected fragment sizes. The vector was transformed into the *Agrobacterium tumefaciens* strain GV3101 by the freeze-thaw method. The strain was verified by PCR, as shown in Figure 2, the fragment was 480, 570, 450, 450, and 704 base pairs (bp), respectively, as expected and then sequenced. It used for transformation when the sequence was correct.

### 2.3. Plant Material and Plant Transformation

A hybrid aspen clone, INRA 353-53 (*P. tremula* × *P. tremuloides*), was used for genetic transformation experiments. Young leaves were cut into 0.5 cm^2^ blocks and placed into a preculture medium, Murashige and Skoog (MS)1 (MS supplemented with 0.5 mg/L 6-benzyladenine (6-BA), 0.2 mg/L naphthaleneacetic acid (NAA), 0.01 mg/L thidiazuron (TDZ), 30 mg/L sugar + 5 mg/L agarose), for three days in the dark. The blocks were then immersed in YEB liquid medium containing 200 μmol/L acetosyringone (AS) with the *Agrobacterium* culture optical density (OD)_600_ = 0.8 for 8 min. The infected leaves were dried on sterile paper and then placed into MS2 medium (MS1 supplemented with 200 μmol/L AS) for another three days in the dark. Next, the wounded leaf disks were transferred onto a selective differentiation medium, MS3 (MS1 supplemented with 50 mg/L kanamycin (KAN) and 500 mg/L cefotaxime (CEF)), for cultivated for 1.5 months at 25 ± 2 °C under a 16 h photoperiod, and the leaves were subcultured bi-weekly. After one month in MS3 medium, resistant shoots ≥ 1.5 cm were cut and transferred to selective rooting medium, MS4 (½ MS supplemented with 0.2 mg/L NAA, 0.2 mg/L indolebutyric acid (IBA), 50 mg/L KAN, 500 mg/L CEF, 30 mg/L sugar and 5 mg/L agarose), for one month and then evaluated by PCR. After subculturing for 1.5 months, transgenic plants were induced to activate the gene-deletor system.

### 2.4. Polymerase Chain Reaction Analysis

Genomic DNA was extracted from glucuronidase gene (GUS)-positive plants using the cetyl trimethyl ammonium bromide (CTAB) method. The GUS-R/GUS-R primers (5′-GCGTTGGC GGTAACAAGAAAGGG, 5′-GGCGCGAAATATTCCCGTGCACC-3′) were used to amplify the 450 bp t-DNA fragment. The PCR parameters were 94 °C for 5 min, 30 cycles at 94 °C for 30 min, 56 °C for 30 s and 72 °C for 1 min, followed by 72 °C for 10 min. The GmHSP-F/GmHSP-R primers (5′-TGGCAGGATATATTGTGGTGTAAACAAATACTAGTACG-3′ and 5′-GTTTACCCGCCAAT ATATCCTGTCAAACACTG-3′) correspond to the 6,125 bp t-DNA sequence. The amplification protocol was performed using the following conditions: 94 °C for 5 min, 35 cycles at 94 °C for 30 min, 62 °C for 30 s and 72 °C for 7 min. The PCR products were separated on a 1.5% agarose gel containing 1% Goldview, and the purified DNA from gels was sequenced for confirmation by Tsingke Environmental Protection Technology Co. Ltd (Beijing, China).

### 2.5. Heat-Shock Induction

Transgenic plants grown in glass jars containing M4 medium were subjected to heat shock at 42 °C for 8 h in a growth chamber under darkness, after which the glass jars were transferred to the culture room as described in [26]. Forty-eight hours after the heat-shock treatment, the explants were analysed by PCR. Non-transgenic plants and plasmids were used as controls.

### 2.6. Southern-Blot Analyses

The restriction enzyme *Eco*RV (Figure 1) was used to digest 20 μg DNA sample at 37 °C overnight, and the fragments were analysed by electrophoresis on a 1% agarose gel. The DNA samples were hybridized to digoxigenin (DIG)-labelled *GUS* probes after transferring to a Hybond N^+^ nylon membrane [27].

### 2.7. Methylation Analyses of the Gmhsp 17.6-L Promoter and FLP Recombinase

Methylation of the *Gmhsp* promoter and *FLP* gene was detected using a bisulphite conversion kit following the manufacturer’s instructions (TsingKe, Beijing, China), using three amplicons in the *Gmhsp* promoter and *FLP* gene. Bisulphite-treated genomic DNA from six transgenic plant lines and sublines was subjected to PCR amplification using the primers shown in Table S3. The DNA sequence data were analysed using the QUMA website (http://quma.cdb.riken.jp/).

### 2.8. Quantitative Real-Time Polymerase Chain Reaction Detection of Gmhsp 17.6-L Promoter and FLP Recombinase

Messenger RNA (mRNA) was isolated from the leaves of transgenic plants before and after heat-shock treatment, according to the manufacturer’s instructions, and then reverse transcribed into first-strand complementary DNA (cDNA). Quantitative real time polymerase chain reaction (qRT-PCR) was performed using actin as the internal control and primers specific for *FLP*. The 10 µL reaction volume contained 0.2 μL each primer, 1 μL cDNA template, 5 µL QuantiFast SYBR Green PCR Master Mix (Qiagen, Hilden, NRW, Germany) and 3.6 μL distillation-distillation H_2_O. Amplification was performed at 95 °C for 5 min, followed by 40 cycles of 95 °C for 10 s, 60 °C for 30 s and 72 °C for 1 min. Standard curves were generated to estimate the reaction efficiency. For each DNA sample, relative target gene and endogene expression was calculated by 2^−ΔΔCt^ [28].

### 2.9. Statistical Analysis

Analysis of variance (ANOVA) was used with Duncan’s test for the statistical analyses, conducted in SPSS version 18.0 (SPPS Inc., Chicago, IL, USA) and Microsoft Excel 2010 (Microsoft Crop., Washington, DC, USA). *p* < 0.05 was considered statistically significant.

## 3. Results

### 3.1. Generation and Characterization of Transgenic Aspen

**Figure 3:**
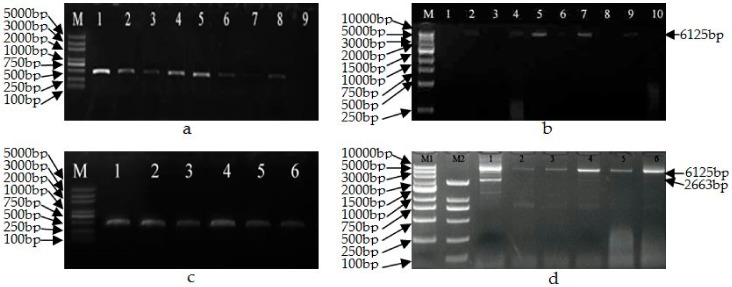
PCR identification of the transgenic plants before (**a**,**b**) and after (**c**,**d**) heat-shock treatment. (**a**) GUSF/GUSR primers. M: Marker 5000; 1–7: transgenic plants; 8: pCaLFGmFNLFG vector; 9: non-transgenic plant. (**b**) GmHSP-F/GmHSP-R primers. M: Marker 10,000; 1: hybrid aspen 353 as control; 2: pCaLFGmFNLFG vector; 4–7, 9, 10: transgenic plants; 3, 8: non-transgenic plant. (**c**) GUSF/GUSR primers. 1–6: transgenic plants after heat-shock treatment. (**d**) GmHSP-F/GmHSP-R primers. M1: Marker 10,000; M2: Marker 2000; 1–6: transgenic plants (1: 22-2-2; 2: 44-3-1; 3: 64-2-1; 4: 64-3-1; 5: 98-3-2; 6: 102-2-2).

Putative transgenic aspen obtained using *Agrobacterium*-mediated transformation methods were verified for transgenicity by PCR using the GUSF/GUSR primers for *gusA* and GmHSP-F/GmHSP-R primers for the t-DNA region. Subsequently, the predicted sizes for partial *GUS* gene (Figure 3a) which showed 450 bp and t-DNA genes which was 6125 bp (Figure 3b) were confirmed in 20 lines. Six transgenic plant lines and their sublines displaying the wild-type phenotype with no morphological variations were treated by heat shock.

### 3.2. Molecular Analysis of Heat-Induced Marker-Free Transgenic Plants

The transgenic plants and their sublines were treated by heat shock for 8 h at 42 °C and analysed by PCR using the GUSF/GUSR and GmHSP-F/GmHSP-R primers. Due to the length of t-DNA, the GUSF/GUSR primer was used to verify transgenic plants; to verify for gene elimination, the GmHSP-F/GmHSP-R primer was used to confirm deletion efficiency (complete, incomplete, or non-complete deletion). The product obtained using GmHSP-F/GmHSP-R primers is predicted to be 2663 bp if deletion occurs and 6125 bp if deletion does not occur, or both 2663 and 6125 bp fragment when incomplete deletion occurred. The results revealed that no complete excision occurred in any of the six lines, which showed a 450 bp fragment (Figure 3c), in the event of complete deletion, there would be no fragment; partial excision was interpreted as partial deletion in some plant cells but not in others, occurred in three lines (lines 22-2-2, 64-3-1 and 102-2-2), which showed 2663 and 6125 bp fragments (Figure 3d), and no deletion occurred in the other three lines (lines 44-3-1, 64-2-1 and 98-3-2), which showed a 6125 bp fragment (Figure 3d).

### 3.3. Analysis of Transgene Copy Number

**Figure 4:**
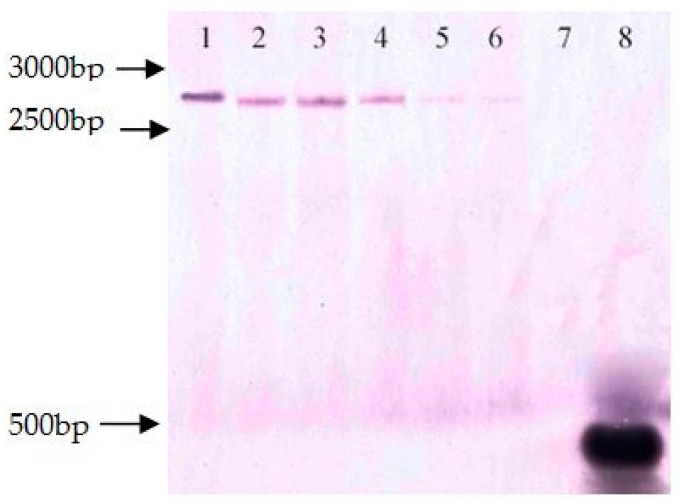
Southern-blot analyses of the *gusA* gene copy number in transgenic plants. 1: 22-2-2; 2: 44-3-1; 3: 64-2-1; 4: 64-3-1; 5: 98-3-2; and 6: 102-2-2; 7: hybrid aspen 353 as control; 8: pCaLFGmFNLFG expression vector.

To explain the excision rate, the six lines were first evaluated by Southern-blot analyses, using a probe specific to *gusA*. As shown in Figure 4, each selected line showed a single copy of *gusA* in the induced six transgenic plants before heat treatment. After heat-shock treatment, among these six lines, partial deletion happened in lines 22-2-2, 64-3-1, and 102-2-2 while no deletion occurred in transgenic clones lines 44-3-1, 64-2-1, and 98-3-2.

### 3.4. Methylation Analysis of the Gmhsp17.6-L and FLP Genes

DNA methylation may impede complete excision of foreign genes in transgenic cells. In the pCaLFGmFNLFG vector, the *Gmhsp* promoter and FLP are important for determining the removal of foreign genes. Therefore, the methylation levels were detected at CpG sites within the *Gmhsp* promoter and *FLP* gene. The analysis included the four transgenic lines 22-2-2, 102-2-2, 44-3-1, 98-3-2, and their sublines which survived and were propagated, among which lines 22-2-2, 102-2-2 showed partial and 44-3-1, 98-3-2 exhibited no deletion after heat-shock treatment. 

**Figure 5:**
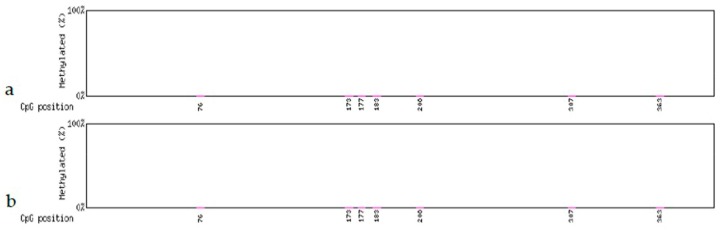
Methylation levels in the *Gmhsp17.6-L* promoter after heat-shock treatment. (**a**) Non-deletion transgenic line 98-3-2. (**b**) Partial-deletion transgenic line 22-2-2.

**Figure 6:**
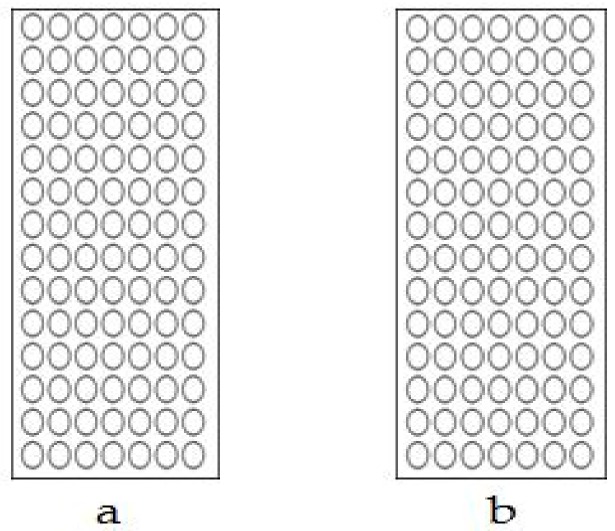
Scatter plot of *Gmhsp17.6-L* methylation after heat-shock treatment. (**a**) Non-deletion transgenic line 98-3-2. (**b**) Partial-deletion transgenic line 22-2-2.

The *Gmhsp* promoter, which contains 397bp bases and seven CpG sites, showed no methylation before, and no change of methylation displayed after treatment. There was no difference between the non-deletion (44-3-1, 98-3-2; Figure 5a and Figure 6a) and partial-deletion (22-2-2; 102-2-2 Figure 5b and Figure 6b) transgenic lines after heat induction. 

**Figure 7:**
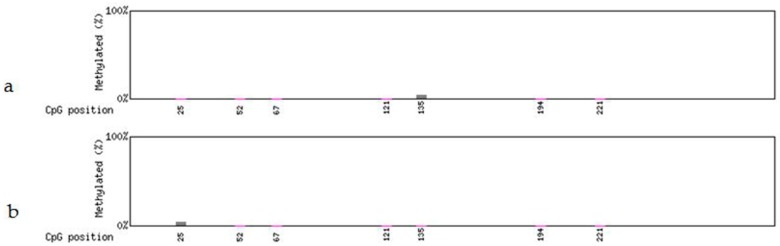
Methylation levels in the partial *FLP* gene sequence after heat-shock treatment. (**a**) Non-deletion transgenic line 98-3-2. (**b**) Partial-deletion transgenic line 22-2-2.

**Figure 8:**
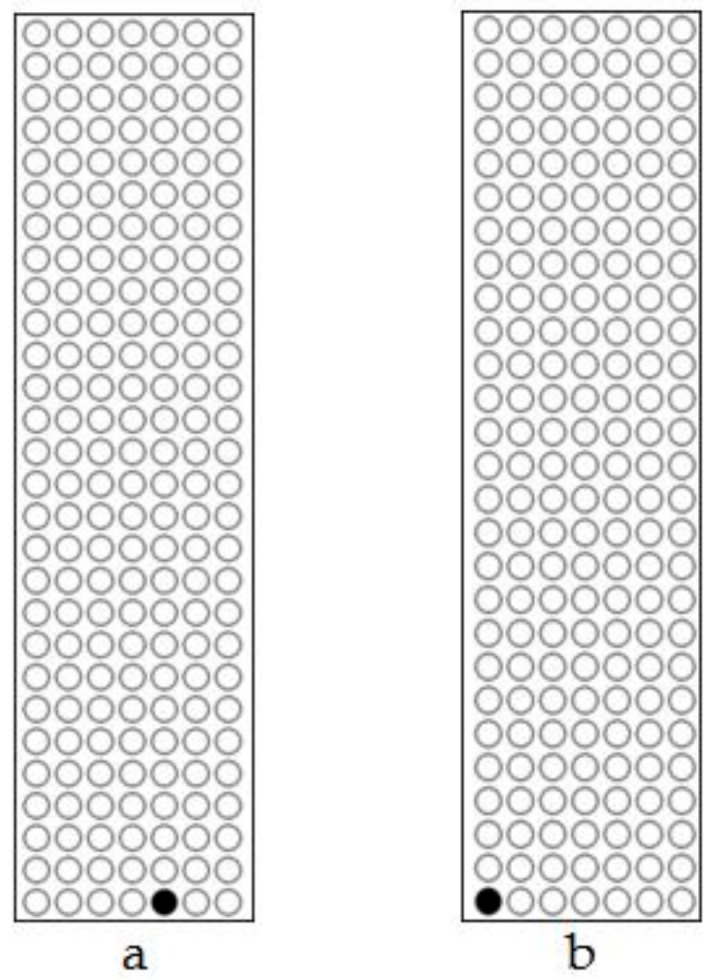
Scatter plot of methylation in the partial *FLP* gene sequence after heat-shock treatment. (**a**) Non-deletion transgenic line 98-3-2. (**b**) Partial-deletion transgenic line 22-2-2.

The analysis of *FLP* recombinase did not show any detectable methylation in the four transgenic lines before heat induction. After treatment methylation was detected in only one of the seven CpG sites of the partial *FLP* sequence (271 bp; Figure S1) in the non-deletion line 98-3-2 (Figure 7a and Figure 8a) and the partial-deletion line 22-2-2 (Figure 7b and Figure 8b), while no methylation was detected in the rest of *FLP* sequence. No *FLP* methylation was detected in the other two lines (44-3-1 and 102-2-2). 

Our results showed no methylation in *Gmhsp17.6-L* and *FLP* before treatment and no difference in methylation after heat shock in the partial- and non-deletion transgenic plants. These indicated that the methylation levels in the *Gmhsp17.6-L* promoter and *FLP* recombinase had no direct relationship with deletion efficiency.

### 3.5. Effect of Heat-Shock Treatment on Gmhsp17.6-L and FLP Gene Expression

The expression of promoter and recombinase is one of factors affecting the deletion efficiency. In our study, the expression levels of the *Gmhsp17.6-L* promoter and *FLP* recombinase in transgenic plant 98-3-2 before heat-shock treatment were used as the control expression levels. 

**Figure 9:**
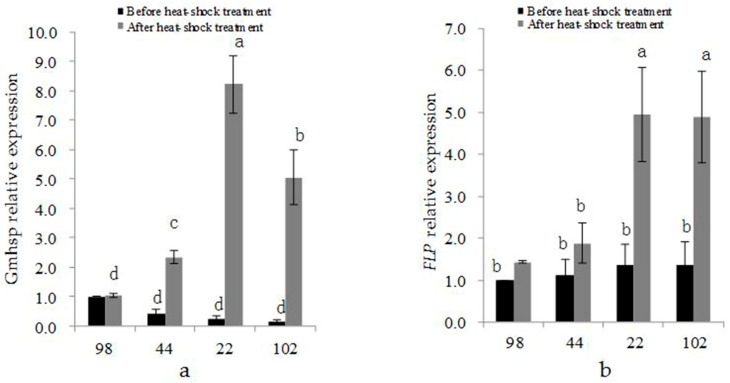
Expression of (**a**) *Gmhsp17.6-L* and (**b**) *FLP* before and after heat-shock treatment in transgenic lines 98-3-2 (98), 44-3-1 (44), 22-2-2 (22), and 102-2-2 (102).

Before heat induction, the expression of *Gmhsp17.6-L* was not different between the partial- and non-deletion transgenic plants. The expression levels of *Gmhsp17.6-L* in the partial-deletion lines 22-2-2 and 102-2-2 were 0.23 and 0.14, respectively, before heat-shock treatment and 8.23 and 5.05, respectively, after treatment, exhibiting more than 30-fold increases (Figure 9a). The expression levels of *Gmhsp17.6-L* in the non-deletion transgenic lines 98-3-2 and 44-3-1 were 1.00 and 0.43, respectively, before heat induction and 1.04 and 2.34, respectively, after heat induction, exhibiting ~1- and ~5- fold increases, respectively. The expression of *Gmhsp17.6-L* was increased more dramatically after heat-shock treatment in the partial-deletion than in the non-deletion plants and the level was significant difference, while no significance presented between two partial- nor non-removal transgenic lines (Figure 9a).

The *FLP* expression level in the partial-deletion and non-deletion lines appeared no significantly different, before heat-shock treatment. While after heat induction, there was significantly higher expression of *FLP* gene in the partial- than in the non-deletion lines. The expression levels of *Gmhsp17.6-L* in the partial-deletion lines 22-2-2 and 102-2-2 were 0.23 and 0.14, respectively, before heat-shock treatment and 8.23 and 5.05, respectively, after treatment, exhibiting more than 30-fold increases. The expression levels of *Gmhsp17.6-L* in the non-deletion transgenic lines 98-3-2 and 44-3-1 were 1.00 and 0.43, respectively, before heat induction and 1.04 and 2.34, respectively, after heat induction, exhibiting ~1- and ~5-fold increase, respectively. No significant difference showed between two partial- nor non-deletion transgenic lines (Figure 9 b).

These results showed that the expression levels of *Gmhsp17.6-L* and *FLP* after heat-shock treatment had an effect on the deletion efficiency. Further higher expression of *Gmhsp17.6-L* and *FLP* promoted the deletion rate in some content.

## 4. Discussion

To delete the marker genes, site-specific recombination systems have been widely applied to Arabidopsis, tobacco, and other crops, and woody plants, often using Cre/*loxP* or Flp/*FRT* methods. Cre/*loxP* was successfully used in Arabidopsis, tobacco, rice, bananas, citrus, and maize [29,30,31,32,33,34]. Flp/*FRT* has been used in grapevine and apples, with non-efficient excision efficiency [35,36]. Compared with Cre/*loxP* and Flp/*FRT* system, the gene-deletor system displays higher excision rate in transgenic tobacco pollen which has shown to be 100% was recently developed [11,12,37]. The system consists of *loxP-FRT* hybrid as recognition site and FLP as recombinase and has been applied to transgenic tobacco plants, *Brassica napus*, rice, petunia, and maize [14,38,39,40,41,42]. In aspen, Cre/*loxP*, Flp/*FRT* and multi-auto-transformation (MAT) vectors are used most frequently [23,43,44]. Until now, no study has employed the gene-deletor system to eliminate marker genes in aspen.

Promoters of the gene-deletor system and site-specific recombination are important factors for removing marker genes in transgenic plants. Among the promoters used, heat-shock promoters are widely utilized due to their controllable and rapid response to heat-shock treatment [45]. Heat-shock promoters from *Drosophila melanogaster*, Arabidopsis, soybean and tobacco have been described previously [21,45,46,47]; in site-specific systems, the former three heat-shock promoters, especially the soybean heat-shock promoter, are widely used [21,24,29,31,34,35,43,48,49]. Until now, there has been no research on using the *Gmhsp17.6-L* promoter in the gene-deletor system. In our experiment, we used the *Gmhsp17.6-L* promoter to control *FLP* recombinase expression in the gene-deletor system.

To activate the heat-shock promoter, the incubation temperature was chosen according to the physiological temperature requirements of plants and the optimum activation temperature for recombinase. Costa et al. [35] used two different temperatures (40 °C, 42 °C) for three heat incubation (1, 2, 3 treatment) to induce transgenic grapevine with Flp*/FRT* system, and found that the removal of marker gene occurred at 40 °C only after the third heat shock, while at 42 °C after the first treatment. Moreover, no significant change happened after three inductions at 42 °C. The study verified that plants growth was not affected after 4 or 6 h induction at 42 °C, however plant growth and vitality was severely affected after 8 h, which coincides to the period required for occurrence of deletion at a window of 6 h or 8 h post induction. Finally the optimized induction for grapevine at 42 °C for 6 h was reported. Herzog et al. [36] showed that the removal rate of marker genes improved with increasing temperature in transgenic apples with Flp/*FRT*, however the increased temperature reduced vitality with an overall optimized induction at 42 °C for 4 h. *Gmhsp17.5-E* was used previously in the Cre/*loxP* and Flp/*FRT* systems in aspen at 42 °C for 6 h [23] or 3 h [50]. *Gmhsp17.6-L* was successfully used at 40 °C to accelerate flowering in aspen INRA 353-53 (male, *P. tremula* × *P. tremuloides*) and INRA 717-1B4 (female, *P. tremula* × *P. alba*) [25]. Taking into account the optimum temperature of recombinase activity and aspen growth in our study, we incubated at 42 °C for 8 h.

There are several factors affecting complete deletion of markers or foreign genes, such as t-DNA copy number, methylation, histone modification, histone variants, small RNAs, and chromatin remodelling [51]. Previous studies have revealed that recombination systems excise more efficiently in transgenic plants with one copy rather than multiple copies t-DNA [18,52,53]. In tobacco, deletion happened mostly in one-copy transgenic lines with the gluthathione-*S*-transferase (GST)-MAT system [18,52]. Similarly, Matsunaga et al. [53] identified that deletion of foreign genes happened in three transgenic poplar using the GST-MAT system in which two out of three lines obtained carried one-copy t-DNA [53]. In addition, Wang et al. [27] studied the transgenic wheat and showed that the deletion rate was affected by the number of marker gene *bar*, and excision occurred in transgenic lines with one or two copy [27]. While Hoenicka et al. [43] studied twelve transgenic poplar which had different copies of CGPDHC::FLP and PTD::FLP-based constructs. The result showed that there was no relationship between t-DNA copy number in transgenic lines and excision efficacy, while in our experiment six transgenic plants used to induce by heat shock were all one-copy, so we couldn’t assess the relationship between t-DNA copy number and efficacy of marker gene excision.

Methylation inhibits the expression of genes, and may affect deletion rate in recombination systems. Hoenicka et al. transformed Cre/*loxP* to poplar and found that the level of methylation was lower in complete- than incomplete-recombination transgenic lines, while they emphasised low levels of methylation could promote but not induce recombination directly [43]. We analysed the effect of methylation on marker gene removal and observed minimal changes in the methylation levels in *Gmhsp17.6-L* and *FLP* by heat shock, which differed from the results of previous study by Hoenicka et al. [43]. 

The expression of foreign genes in transgenic plants changed when plants were under induction and could affect the excision efficiency in transgenic lines with recombination system. Éva et al. obtained transgenic barley with Cre/*loxP* system and verified that Cre could accumulate massively in low but be active in higher temperature, low temperature inhibited the premature deletion of the Cre encoding gene, and promote high expression of recombinase therefore increased excision frequency when temperature was high enough to induce Cre enzyme activity [54]. Our results suggest that a high temperature improves the activation of *Gmhsp17.6-L* to induce the expression of *FLP* in order to recognize the *loxP-FRT* locus and to excise foreign genes between two *loxP*-*FRT* sites. Incomplete excision occurs possibly because the recombinase activity is affected by gene transcription, temperature, expression levels [54], or insufficient expression of recombinase, different sensitiveness of plant tissues [35]. 

In our study, the gene-deletor system with the promoter *Gmhsp17.6-L* was used in transgenic aspen with three transgenic plants with partial excision and three plants with no excision.

There was no direct relationship of t-DNA copy number or *Gmhsp17.6-L* and *FLP* methylation levels with excision efficacy, while the expression of *Gmhsp17.6-L* promoter and *FLP* recombinant enzyme affected the excision efficiency which was improved by the increased expression level of *Gmhsp17.6-L* promoter and *FLP* recombinase in some content. These results validate this system and provide empirical support for its use in other trees. However, more work needs to be done, such as improving excision rates and constructing gene-deletor systems containing other promoters to satisfy various need.

## Supplementary Materials

The following are available online at http://www.mdpi.com/2073-4425/9/10/484/s1, Table S1: The primers for constructing pCaLFGmFNLFG vector, Table S2: The primers for verifying pCaLFHFGNLF vector, Table S3: The primers for methylation, Figure S1: The sequence of partial *FLP* gene.

## References

[1] Fillatti J.A.J., Sellmer J., Mccown B., Haissig B., Comai L. (1987). *Agrobacterium* mediated transformation and regeneration of *Populus*.. Mol. Gen. Genet..

[2] Klocko A.L., Meilan R., James R.R., Viswanath V., Ma C., Payne P., Miller L., Skinner J.S., Oppert B., Cardineau G.A. (2014). *Bt-Cry3Aa* transgene expression reduces insect damage and improves growth in field-grown hybrid poplar. Can. J. For. Res..

[3] Karim A., Jiang Y., Guo L., Ling Z., Ye S., Duan Y., Li C., Luo K. (2015). Isolation and characterization of a subgroup IIa WRKY transcription factor PtrWRKY40 from *Populus trichocarpa*.. Tree Physiol..

[4] Wilkerson C.G., Mansfield S.D., Lu F., Withers S., Park J.Y., Karlen S.D., Gonzales-Vigil E., Padmakshan D., Unda F., Rencoret J. (2014). Monolignol ferulate transferase introduces chemically labile linkages into the lignin backbone. Science.

[5] Komari T., Hiei Y., Saito Y., Mural N., Kumashiro T. (1996). Vectors carrying two separate t-DNAs for cotransformation of higher plants mediated by *Agrobacterium tumefaciens* and segregation of transformants free from selection markers. Plant J..

[6] Zubko E., Scutt C., Meyer P. (2000). Intrachromosomal recombination between attP regions as a tool to remove selectable marker genes from tobacco transgenes. Nat. Biotechnol..

[7] Cotsaftis O., Sallaud C., Breitler J.C., Meynard D., Greco R., Pereira A., Guiderdoni E. (2002). Transposon-mediated generation of t-DNA- and marker-free rice plants expressing a *Bt* endotoxin gene. Mol. Breed..

[8] Goldsbrough A.P., Lastrella C.N., Yoder J.I. (1993). Transposition mediated re–positioning and subsequent elimination of marker genes from transgenic tomato. Nat. Biotechnol..

[9] Fladung M., Nowitzki O., Kumar S., Hoenicka H. (2005). The site-specific recombination systems Cre-*lox* and FLP-*FRT* are functionally active in poplar. For. Genet..

[10] Nanto K., Ebinuma H. (2008). Marker-free site-specific integration plants. Transgenic Res..

[11] Luo K., Duan H., Zhao D., Zheng X., Deng W., Chen Y., Jiang X., McAvoy R., Wu Y., Pei Y. (2007). ‘GM-gene-deletor’: Fused loxP-*FRT* recognition sequences dramatically improve the efficiency of FLP or CRE recombinase on transgene excision from pollen and seed of tobacco plants. Plant Biotechnol. J..

[12] Li Y., Duan H., Smith W. (2007). Gene-Deletor System: A New Tool to Address Concerns over GE Crops.

[13] Zhao D., Lv L., He A., Luo K., Duan H., Zheng X., Deng W., Chen Y., An X., He M. (2008). The gene-deletor technology: Principle and potential application in genetically engineered agriculture. Mol. Plant Breed..

[14] Lu L., Liu Y., Zhu Y., Zhao D. (2010). Selectable gene auto-excision via a cold inducible ‘gene deletor’ system. Afr. J. Agric. Res..

[15] Li Y. (2012). Gene deletor: A new tool to address gene flow and food safety concerns over transgenic crop plants. Front. Biol..

[16] Mlynárová L., Conner A.J., Nap J.P. (2006). Directed microspore-specific recombination of transgenic alleles to prevent pollen-mediated transmission of transgenes. Plant Biotechnol. J..

[17] Moravčíková J., Vaculková E., Bauer M., Libantová J. (2008). Feasibility of the seed specific cruciferin C promoter in the self-excision Cre/*loxP* strategy focused on generation of marker-free transgenic plants. Theor. Appl. Genet..

[18] Sugita K., Kasahara T., Matsunaga E., Ebinuma H. (2000). A transformation vector for the production of marker-free transgenic plants containing a single copy transgene at high frequency. Plant J..

[19] Sreekala C., Wu L., Gu K., Wang D., Tian D., Yin Z. (2005). Excision of a selectable marker in transgenic rice (*Oryza sativa* L.) using a chemically regulated Cre/*loxP* system. Plant Cell Rep..

[20] Zhang Y., Li H., Ouyang B., Lu Y., Ye Z. (2006). Chemical-induced autoexcision of selectable markers in elite tomato plants transformed with a gene conferring resistance to lepidopteran insects. Biotechnol. Lett..

[21] Kilby N.J., Davies G.J., Snaith M.R., Murray J.A.H. (1995). FLP recombinase in transgenic plants: Constitutive activity in stably transformed tobacco and generation of marked cell clones in *Arabidopsis*.. Plant J..

[22] Hoff T., Schnorr K.M., Mundy J. (2001). A recombinase-mediated transcriptional induction system in transgenic plants. Plant Mol. Biol..

[23] Fladung M., Becker D. (2010). Targeted integration and removal of transgenes in hybrid aspen (*Populus tremula* L. x *P. tremuloides* Michx.) using site-specific recombination systems. Plant Biology.

[24] Chong-Pérez B., Kosky R.G., Reyes M., Rojas L., Ocaña B., Tejeda M., Pérez B., Angenon G. (2012). Heat shock induced excision of selectable marker genes in transgenic banana by the Cre-*lox* site-specific recombination system. J. Biotechnol..

[25] Zhang H., Harry D.E., Ma C., Yuceer C., Hsu C.Y., Vikram V., Shevchenko O., Etherington E., Strauss S.H. (2010). Precocious flowering in trees: the *FLOWERING LOCUS T* gene as a research and breeding tool in *Populus*.. J. Exp. Bot..

[26] Zhang Y., Wang B., Guo L., Xu W., Wang Z., Li B., Zhang J. (2017). Factors influencing direct shoot regeneration from leaves, petioles, and plantlet roots of triploid hybrid *Populus* sect. *Tacamahaca*.. J. For. Res..

[27] Wang K., Liu H., Du L., Ye X. (2017). Generation of marker-free transgenic hexaploid wheat via an *Agrobacterium*-mediated co-transformation strategy in commercial Chinese wheat varieties. Plant Biotechnol. J..

[28] Livak K.J., Schmittgen T.D. (2001). Analysis of relative gene expression data using real-time quantitative PCR and the 2^−ΔΔCT^ method. Methods.

[29] Rao M.R., Moon H.S., Schenk T.M., Becker D., Mazarei M., Stewart C.N. (2010). FLP/*FRT* recombination from yeast: Application of a two gene cassette scheme as an inducible system in plants. Sensors.

[30] Zheng Y., Pan Y., Li J., Zhou Y., Pan Y., Ding Y., Su C., Zhang X. (2016). Visible marker excision via heat-inducible Cre/*LoxP* system and *Ipt* selection in tobacco. In Vitro Cell. Dev. Pl..

[31] Khattri A., Nandy S., Srivastava V. (2011). Heat-inducible Cre-*lox* system for marker excision in transgenic rice. J. Biosci..

[32] Chong-Pérez B., Reyes M., Rojas L., Ocaña B., Ramos A., Kosky R.G., Angenon G. (2013). Excision of a selectable marker gene in transgenic banana using a Cre/*lox* system controlled by an embryo specific promoter. Plant Mol. Biol..

[33] Zou X., Peng A., Xu L., Liu X., Lei T., Yao L., He Y., Chen S. (2013). Efficient auto-excision of a selectable marker gene from transgenic citrus by combining the Cre/*loxP* system and *ipt* selection. Plant Cell Rep..

[34] Zhang W., Subbarao S., Addae P., Shen A., Armstrong C., Peschke V., Gilbertson L. (2003). Cre/*lox*-mediated marker gene excision in transgenic maize (*Zea mays* L.) plants. Theor. Appl. Genet..

[35] Costa L.D., Piazza S., Campa M., Flachowsky H., Hanke M.V., Malnoy M. (2016). Efficient heat-shock removal of the selectable marker gene in genetically modified grapevine. Plant Cell Tissue Organ Cult..

[36] Herzog K., Flachowsky H., Deising H.B., Hanke M.V. (2012). Heat-shock-mediated elimination of the *nptII* marker gene in transgenic apple (*Malus* × *domestica* Borkh.). Gene.

[37] Ding J., Duan H., Deng Z., Zhao D., Yi G., McAvoy R., Li Y. (2014). Molecular strategies for addressing gene flow problems and their potential applications in abiotic stress tolerant transgenic plants. Crit. Rev. Plant Sci..

[38] Qin L., Zhao D., Zhao D. (2015). Overexpression of *NrCN* improved TMV resistance in selection marker-free tobacco generated by Gene-Deletor system. Plant Mol. Biol. Rep..

[39] Xu M. (2009). The Application of the Gene-Deletor Technology in Transgenic *Brassica napus*.. Master’s Thesis.

[40] Zhu Y. (2010). The Preliminary Study on the Construction of Exogenous Gene Specific. Master’s Thesis.

[41] Li Y., Zhao D. (2011). Genetic transformation efficiency enhanced by *ipt* gene and heat shock promoter driven gene excision in petunia. Genom. Appl. Biol..

[42] Xiang Y., Liu Y., Zhao D., Li Y. (2015). Application of gene-deletor system to create multiple stress tolerant corn new germplasm with gene *ZmSDD1*, *BT* and *BAR*.. Mol. Plant Breed..

[43] Hoenicka H., Lehnhardt D., Nunna S., Reinhardt R., Jeltsch A., Briones V., Fladung M. (2016). Level of tissue differentiation influences the activation of a heat-inducible flower-specific system for genetic containment in poplar (*Populus tremula* L.). Plant Cell Rep..

[44] Zelasco S., Ressegotti V., Confalonieri M., Carbonera D., Calligari P., Bonadei M., Bisoffi S., Yamada K., Balestrazzi A. (2007). Evaluation of MAT-vector system in white poplar (*Populus alba* L.) and production of *ipt* marker-free transgenic plants by ‘single-step transformation’. Plant Cell Tissue Organ Cult..

[45] Navarre C., Sallets A., Gauthy E., Maîtrejean M., Magy B., Nader J., Thozée C., Crouzet J., Batoko H., Boutry M. (2011). Isolation of heat shock-induced *Nicotiana tabacum* transcription promoters and their potential as a tool for plant research and biotechnology. Transgenic Res..

[46] Ashburner M., Bonner J.J. (1979). The induction of gene activity in drosophila by heat shock. Cell.

[47] Takahashi T., Komeda Y. (1989). Characterization of two genes encoding small heat-shock proteins in *Arabidopsis thaliana*.. Mol. Gen. Genet..

[48] Fladung M., Polak O. (2012). *Ac*/Ds-transposon activation tagging in poplar: A powerful tool for gene discovery. BMC Genom..

[49] Wang Y., Chen B., Hu Y., Li J., Lin Z. (2005). Inducible excision of selectable marker gene from transgenic plants by the Cre/*lox* site-specific recombination system. Transgenic Res..

[50] Fladung M., Schenk T.M.H., Polak O., Becker D. (2010). Elimination of marker genes and targeted integration via FLP/*FRT* recombination system from yeast in hybrid aspen (*Populus tremula* L. × *P. tremuloides* Michx.). Tree Genet. Genomes.

[51] Ahmad A., Zhang Y., Cao X.-F. (2010). Decoding the epigenetic language of plant development. Mol. Plant.

[52] Endo S., Kasahara T., Sugita K., Ebinuma H. (2002). A new GST-MAT vector containing both *ipt* and *iaaM/H* genes can produce marker-free transgenic tobacco plants with high frequency. Plant Cell Rep..

[53] Matsunaga E., Sugita K., Ebinuma H. (2002). Asexual production of selectable marker-free transgenic woody plants, vegetatively propagated species. Mol. Breed..

[54] Éva C., Téglás F., Zelenyánszki H., Tamás C., Juhász A., Mészáros K., Tamás L. (2018). Cold inducible promoter driven Cre-*lox* system proved to be highly efficient for marker gene excision in transgenic barley. J. Biotechnol..

