# Supplementary material for: Heat-Shock-Induced Removal of Transgenes Using the Gene-Deletor System in Hybrid Aspen (Populus tremula × P. tremuloides)
Source: Genes (Basel). 2018 Oct 8;9(10):484. doi: 10.3390/genes9100484 (PMC6210648; doi:10.3390/genes9100484)
Supplement: Supplementary file 1 [file genes-09-00484-s001.pdf]

Table S1: The primers for constructing pCaLFGmFNLFG vector

The primers	The sequences of primers
III-NOS-FF	AGGCAAACAAGAGCTCGGTACCGATCGTTCAAACATTTGG
III-NOS-RR	GCAGGCGGCCGCCTTAGATCTAGTAACATAGA
III-RB-FF	TAAGGCGGCCGCCTGCAGAACTATCAGTGTTTGAC
III-RB-R	TAAGCGCATGCACATACAAATGGACGA
Gmhsp-9	ATATTTTTCTGGAACATACAAGAGTATCCTTTCACTTCCTTTAAATACC
III-XhoI-R	CATTTTTGGAGTAGGGGACAAAAAGAACCGCATA
III-35S-FFF	tatgCGGTTCTTTTTGTCCCCTACTCCAAAAATG
III-35S-RR	CCATCCATTGTCCTCTCCAAATGAA
III-NPT-FF	CATTTGGAGAGGACAATGGATGGATTGCACGCAGG
III-LOXP-RR	TGACATTTTTGGAGTAGGGGGGATCCAAGTTCCTATTCCGAAG

Table S2: The primers for verificating pCaLFHFGNLF vector

The primers	The sequences of primers
GMHSP-F	TGGGCCACAAAACGTATAGATCA
GMHSP-R	TTCCACAAACTGACGAACAAGCA
F3t	AATAATCAGGAAGTGATGGAGCA3
R3t	CGACCAAAGCCAGTAAAGTAGAA3
Fmid	TCCAGTGTTTAGTGACAGAGACA
Rmid	AGTGCGAAGTAGTGATCAGGTAT
gus-nptII-F	GCGTTGGCGGTAACAAGAAAGGG
gus-nptII-R	CAGGAGCAAGGTGAGATGACAGGAGAT
FLP-35SF	GGAGTTGACTAATGTTGTGGGAAAT
FLP-35SR	GACAGATAGCTGGGCAATGGAAT

Table S3: The primers for methylation

The primers	The sequences of primers
GMHSP-F1	GATTGAAGTTTTTATATTTTTTAGAG
GMHSP-R2	TCCACAAACTAACGAACAAACACC
GMHSP-R1	TTCTCACCTAAAAATCTTTCAAACC
GMHSP-F2	GAAGTTTTTATATTTTTTAGAGAATAGG
FLP-1F1	TGATATTATTAAATAAGAGAAGAGT
FLP-1R2	ATCAATTTCTTTAATAAAACTTCCA
FLP-1R1	AAAAATAATTATAAATTCCCAAAC
FLP-2F1	GTAAAAAGTAATAATTTTGGAAGT
FLP-2R2	CTTATCTTTATCTCTATCACT
FLP-2R1	CTAAAAAAATATATATACCTAC
FLP-3F2	TATTTGGGAGTAATAATTTAGTG
FLP-3F1	TTAGTTTAAAATAAGTATTTGGGAG
FLP-3R1	TTATCCTAACCACGACAAAAACAC
FLP-3R2	CCCACAACATTAATCAACTCC
FLP-4F1	GATTTTATTTTTTTAATGAAGG
FLP-4R	CAATCTTAAAAAACTTTATTACC
FLP-4F2	GGAGTTGATTAATGTTGTGGG

TTTAGTGACAGAGACAAAGACAAGCGTTAGTAGGCACATATACTTCTTTA
GCGCAAGGGGTAGGATCGATCCACTTGTATATTTGGATGAATTTTTGAGG
AATTCTGAACCAGTCCTAAAACGAGTAAATAGGACCGGCAATTCTTCAAG
CAATAAACAGGAATACCAATTATTAAAAGATAACTTAGTCAGATCGTACA
ATAAAGCTTTGAAGAAAAATGCGCCTTATTCAATCTTTGCTATAAAAAAT
GGCCCAAAATCTCACATTGGAAGACATTTGATGACCTCATTTCTTTCAAT
GAAGGGCCTAAC

Figure S1: The sequence of partial *FLP* gene
